# Supplementary material for: Morphofunctional Assessment beyond Malnutrition: Fat Mass Assessment in Adult Patients with Phenylketonuria—Systematic Review
Source: Nutrients. 2024 Jun 11;16(12):1833. doi: 10.3390/nu16121833 (PMC11206948; doi:10.3390/nu16121833)
Supplement: Supplementary file 1 [file nutrients-16-01833-s001.zip › nutrients-3031492-supplementary.pdf]

Supplementary materials

S1. Search details
Databases:
- Pubmed
- EMBASE

A combination was used of:
- MESH (Medical Subject Headings) terms from Pubmed and Emtree terms from EMBASE
- Text-word search in all fields

Entry terms:
1. Fat mass
2. Bioimpedance
3. Computed Tomography
4. Magnetic Resonance Imaging
5. Plethysmography
6. Dual X-ray Absorptiometry (DXA)
7. Ultrasound
8. Inborn errors of metabolism
9. Phenylketonuria
10. (1 OR 2 OR 3 OR 4 OR 5 OR 6 OR 7)
11. 10. AND 8.
12. 11. Limit "Adult"
13. 10. AND 9.
14. 13. Limit "Adult"
15. 1. AND 9.
16. 15. Limit "Adult"

S2. List of excluded studies and reasons

S2.1. Articles that did not provide information relevant to PECO outcome (21).

1. Aldámiz-Echevarría, L.; Bueno, M.A.; Couce, M.L.; Lage, S.; Dalmau, J.; Vitoria, I.; Andrade, F.; Blasco, J.; Alcalde, C.; Gil, D.; et al. Anthropometric characteristics and nutrition in a cohort of PAH-deficient patients. *Clin Nutr*. **2014**, *33* (4), 702-17. doi: https://www.doi.org/10.1016/j.clnu.2013.09.011

2. Burrage, L.C.; McConnell, J.; Haesler, R.; O'Riordan, M.A.; Sutton, V.R.; Kerr, D.S.; McCandless, S.E. High prevalence of overweight and obesity in females with phenylketonuria. Mol Genet Metab. 2012 Sep;107(1-2):43-8. doi: https://www.doi.org/10.1016/j.ymgme.2012.07.006

3. Camatta, G.C.; Kanufre, V.C.; Alves, M.R.A.; Soares, R.D.L.; Norton, R.C.; de Aguiar, M.J.B.; Starling, A.L.P. Body fat percentage in adolescents with phenylketonuria and associated factors. *Mol Genet Metab Rep*. **2020**, 23, 100595. doi: https://www.doi.org/10.1016/j.ymgmr.2020.100595

4. Couce, M.L.; Vitoria, I.; Aldámiz-Echevarría, L.; Fernández-Marmiesse, A.; Roca, I.; Llarena, M.; Sánchez-Pintos, P.; Leis, R.; Hermida, A. Lipid profile status and other related factors in patients with Hyperphenylalaninaemia. *Orphanet J Rare Dis*. **2016**, *11* (1), 123. doi: https://www.doi.org/10.1186/s13023-016-0508-x

5. Daly, A.; Evans, S.; Pinto, A.; Jackson, R.; Ashmore, C.; Rocha, J.C.; MacDonald, A. The Impact of the Use of Glycomacropeptide on Satiety and Dietary Intake in Phenylketonuria. *Nutrients*. **2020**, *12* (9), 2704. doi: https://www.doi.org/10.3390/nu12092704

6. Dios-Fuentes, E.; Gonzalo Marin, M.; Remón-Ruiz, P.; Benitez Avila, R.; Bueno Delgado, M.A.; Blasco Alonso, J.; Doulatram Gamgaram, V.K.; Olveira, G.; Soto-Moreno, A.; Venegas-Moreno, E. Cardiometabolic and Nutritional Morbidities of a Large, Adult, PKU Cohort from Andalusia. *Nutrients*. **2022**, *14* (6), 1311. doi: https://www.doi.org/10.3390/nu14061311

7. Enns, G.M.; Koch, R.; Brumm, V.; Blakely, E.; Suter, R.; Jurecki, E. Suboptimal outcomes in patients with PKU treated early with diet alone: revisiting the evidence. *Mol Genet Metab*. **2010**, *101* (2-3), 99-109. doi: https://www.doi.org/10.1016/j.ymgme.2010.05.017

8. Gokmen Ozel, H.; Ahring, K.; Bélanger-Quintana, A.; Dokoupil, K.; Lammardo, A.M.; Robert, M.; Rocha, J.C.; Almeida, M.F.; van Rijn, M.; MacDonald, A. Overweight and obesity in PKU: The results from

8 centres in Europe and Turkey. *Mol Genet Metab Rep*. **2014**, *1*, 483-486. doi: https://www.doi.org/10.1016/j.ymgmr.2014.11.003

9. Gramer, G.; Haege, G.; Langhans, C.D.; Schuhmann, V.; Burgard, P.; Hoffmann, G.F. Long-chain polyunsaturated fatty acid status in children, adolescents and adults with phenylketonuria. *Prostaglandins Leukot Essent Fatty Acids*. **2016**, *109*, 52-7. doi: https://www.doi.org/10.1016/j.plefa.2016.04.005

10. Hochuli, M.; Bollhalder, S.; Thierer, C.; Refardt, J.; Gerber, P.; Baumgartner, M.R. Effects of Inadequate Amino Acid Mixture Intake on Nutrient Supply of Adult Patients with Phenylketonuria. *Ann Nutr Metab*. **2017**, *71* (3-4), 129-135. doi: https://www.doi.org/10.1159/000479746

11. Htun, P.; Nee, J.; Ploeckinger, U.; Eder, K.; Geisler, T.; Gawaz, M.; Bocksch, W.; Fateh-Moghadam, S. Fish-Free Diet in Patients with Phenylketonuria Is Not Associated with Early Atherosclerotic Changes and Enhanced Platelet Activation. *PLoS One*. **2015**, *10* (8), e0135930. doi: https://www.doi.org/10.1371/journal.pone.0135930

12. Leiva, C.; Bravo, P.; Arias, C.; Cabello, J.F.; Leal-Witt, M.J.; Salazar, F.; Cornejo, V. 25 Hydroxy Vitamin D Level, Bone Health, Vitamin D and Calcium Intake in Chilean Patients with Phenylketonuria and Hyperphenylalaninemias. *J inborn errors metab screen* [Internet]. **2021**, *9*, e20210004. Available from: https://www.doi.org/10.1590/2326-4594-JIEMS-2021-0004

13. MacLeod, E.L.; Gleason, S.T.; van Calcar, S.C.; Ney, D.M. Reassessment of phenylalanine tolerance in adults with phenylketonuria is needed as body mass changes. *Mol Genet Metab*. **2009**, *98* (4), 331-7. doi: https://www.doi.org/10.1016/j.ymgme.2009.07.016

14. Mazzola, P.N.; Teixeira, B.C.; Schirmbeck, G.H.; Reischak-Oliveira, A.; Derks, T.G.J.; van Spronsen, F.J.; Dutra-Filho, C.S.; Schwartz, I.V.D. Acute exercise in treated phenylketonuria patients: Physical activity and biochemical response. *Mol Genet Metab Rep*. **2015**, *5*, 55-59. doi: https://www.doi.org/10.1016/j.ymgmr.2015.10.003

15. Ney, D.M.; Stroup, B.M.; Clayton, M.K.; Murali, S.G.; Rice, G.M.; Rohr, F.; Levy, H.L. Glycomacropeptide for nutritional management of phenylketonuria: a randomized, controlled, crossover trial. *Am J Clin Nutr*. **2016**, *104* (2), 334-45. doi: https://www.doi.org/10.3945/ajcn.116.135293

16. Okano, Y.; Hattori, T.; Fujimoto, H.; Noi, K.; Okamoto, M.; Watanabe, T.; Watanabe, R.; Fujii, R.; Tamaoki, T. Nutritional status of patients with phenylketonuria in Japan. *Mol Genet Metab Rep*. **2016**, *8*, 103-10. doi: https://www.doi.org/10.1016/j.ymgmr.2016.08.005

17. Rodrigues, C.; Pinto, A.; Faria, A.; Teixeira, D.; van Wegberg, A.M.J.; Ahring, K.; Feillet, F.; Calhau, C.; MacDonald, A.; Moreira-Rosário, A.; Rocha, J.C. Is the Phenylalanine-Restricted Diet a Risk Factor for Overweight or Obesity in Patients with Phenylketonuria (PKU)? A Systematic Review and Meta-Analysis. *Nutrients*. **2021**, *13* (10), 3443. doi: https://www.doi.org/10.3390/nu13103443

18. van Calcar, S.C.; MacLeod, E.L.; Gleason, S.T.; Etzel, M.R.; Clayton, M.K.; Wolff, J.A.; Ney, D.M. Improved nutritional management of phenylketonuria by using a diet containing glycomacropeptide compared with amino acids. *Am J Clin Nutr*. **2009**, *89* (4), 1068-77. doi: https://www.doi.org/10.3945/ajcn.2008.27280

19. Vazquez-Agra, N.; Fernandez-Crespo, S.; Marques-Afonso, A.T.; Cruces-Sande, A.; Barbosa-Gouveia, S.; Martinez-Olmos, M.A.; Hermida-Ameijeiras, A. The correlation of lipid profile and waist circumference with phenylalanine levels in adult patients with classical phenylketonuria. *Med Clin (Barc)*. **2023**, *160* (9), 385-391. English, Spanish. doi: https://www.doi.org/10.1016/j.medcli.2022.09.025

20. Viau, K.; Wessel, A.; Martell, L.; Sacharow, S.; Rohr, F. Nutrition status of adults with phenylketonuria treated with pegvaliase. *Mol Genet Metab*. **2021**, *133* (4), 345-351. doi: https://www.doi.org/10.1016/j.ymgme.2021.06.002

21. Wernlund, P.G.; Hvas, C.L.; Dahlerup, J.F.; Bahl, M.I.; Licht, T.R.; Knudsen, K.E.B.; Agnholt, J.S. Casein glycomacropeptide is well tolerated in healthy adults and changes neither high-sensitive C-reactive protein, gut microbiota nor faecal butyrate: a restricted randomised trial. *Br J Nutr*. **2021**, *125* (12), 1374-1385. doi: https://www.doi.org/10.1017/S0007114520003736

S2.2. Articles in which sample was exclusively or mainly pediatric (5).

1. de Castro, M.J.; Sánchez-Pintos, P.; Abdelaziz-Salem, N.; Leis, R.; Couce, M.L. Evaluation of Body Composition, Physical Activity, and Food Intake in Patients with Inborn Errors of Intermediary Metabolism. *Nutrients*. **2021**, *13* (6), 2111. doi: https://www.doi.org/10.3390/nu13062111

2. Doulgeraki, A.; Skarpalezou, A.; Theodosiadou, A.; Monopolis, I.; Schulpis, K. Body composition profile of young patients with phenylketonuria and mild hyperphenylalaninemia. *Int J Endocrinol Metab*. **2014**, *12* (3), e16061. doi: https://www.doi.org/10.5812/ijem.16061

3. Evans, M.; Truby, H.; Boneh, A. The relationship between dietary intake, growth and body composition in Phenylketonuria. *Mol Genet Metab*. **2017**, *122* (1-2), 36-42. doi: https://www.doi.org/10.1016/j.ymgme.2017.07.007

4. Evans, M.; Nguo, K.; Boneh, A.; Truby H. The Validity of Bioelectrical Impedance Analysis to Measure Body Composition in Phenylketonuria. *JIMD Rep*. **2018**, *42*, 37-45. doi: https://www.doi.org/10.1007/8904_2017_75

5. Mazzola, P.N.; Nalin, T.; Castro, K.; van Rijn, M.; Derks, T.G.; Perry, I.D.; Mainieri, A.S.; Schwartz, I.V. Analysis of body composition and nutritional status in Brazilian phenylketonuria patients. *Mol Genet Metab Rep*. **2016**, *6*, 16-20. doi: https://www.doi.org/10.1016/j.ymgmr.2015.12.003

S2.3. Article that did not include patients with PKU but healthy volunteers (1).

1. Alfheeaid, H.; Gerasimidis, K.; Năstase, A.M.; Elhauge, M.; Cochrane, B.; Malkova, D. Impact of phenylketonuria type meal on appetite, thermic effect of feeding and postprandial fat oxidation. *Clin Nutr*. **2018**, *37* (3), 851-857. doi: https://www.doi.org/10.1016/j.clnu.2017.03.005

S2.4. Article with fat mass results in a PKU sample already reported (1).

1. Rocha, J.C.; van Spronsen, F.J.; Almeida, M.F.; Ramos, E.; Guimarães, J.T.; Borges, N. Early dietary treated patients with phenylketonuria can achieve normal growth and body composition. *Mol Genet Metab*. **2013**, *110* Suppl, S40-3. doi: https://www.doi.org/10.1016/j.ymgme.2013.10.009

S3. Figure S1.

	Question / objective	Definition of study population	Elegible persons	Inclusion & exclusion criteria	Sample size justification	Meaurement of outcome	Blinding of outcome assessors	Rate of follow-up	Adjustement of confounding variables	Other
Alghamdi 2021	+	-	nr	-	-	+	-	na	-	
Barta 2022	+	+	nr	+	-	+	-	na	+	
Jani 2017	+	+	nr	+	+	+	-	na	+	No control group, reference U.S. population
Mezzomo 2023	+	+	-	+	+	+	-	na	-	
Montanari 2022	+	-	nr	+	-	+	-	+	-	No control group
Rocha 2012	+	+	nr	+	+	+	-	na	+	
Rojas Agurto 2023	+	+	nr	+	+	+	-	na	+	
Stroup 2018	+	+	+	+	+	+	-	na	+	No control group
Weng 2020	-	-	nr	-	-	+	-	na	-	
Zerjav Tansek 2020	+	+	nr	-	-	+	-	na	+	No healthy control group, retrospective

Figure S1. Summary of the risk of bias of every reviewed study including every selected item from the "Guidance for assessing the quality of cohort and cross-sectional studies".

National Institutes of Health. Quality Assessment Tool for Observational Cohort and Cross-Sectional Studies. Available online: https://www.nhlbi.nih.gov/health-topics/study-quality-assessment-tools (accessed on April 1st, 2024).

Green (+): low risk of bias; red (-): high risk of bias nr: not reported; na: not applicable.
